# Effects of a Pair Programming Educational Robot-Based Approach on Students’ Interdisciplinary Learning of Computational Thinking and Language Learning

**DOI:** 10.3389/fpsyg.2022.888215

**Published:** 2022-05-24

**Authors:** Ting-Chia Hsu, Ching Chang, Long-Kai Wu, Chee-Kit Looi

**Affiliations:** ^1^Department of Technology Application and Human Resource Development, National Taiwan Normal University, Taipei City, Taiwan; ^2^Faculty of Artificial Intelligence in Education, Central China Normal University, Wuhan, China; ^3^National Institute of Education, Nanyang Technological University, Singapore, Singapore

**Keywords:** interdisciplinary activities, educational robots, pair programming, language learning, trial-and-error loops

## Abstract

Using educational robots (ERs) to integrate computational thinking (CT) with cross-disciplinary content has gone beyond Science, Technology, Engineering, and Mathematics (STEM), to include foreign-language learning (FL) and further cross-context target-language (TL) acquisition. Such integration must not solely emphasise CT problem-solving skills. Rather, it must provide students with interactive learning to support their target-language (TL) interaction while reducing potential TL anxiety. This study aimed to validate the effects of the proposed method of pair programming (PP) along with question-and-response interaction in a board-game activity on young learners’ CT skills and TL learning across contexts. Two Grade 6 classes, one with 16 students who were studying Chinese as a Second Language (CSL) and the other with 16 students who were studying English as a Foreign Language (EFL), participated in the activity. A series of instruments on achievement assessment, questionnaires on CT skills and TL anxiety, and sequential learning behaviour analysis were used to critically examine the results. The main conclusion is that the EFL group showed better social skills of cooperation on CT and lower TL learning anxiety, while the CSL group demonstrated better problem-solving skills in CT, but presented more behaviours of trial-and-error loops. Results not only contribute suggestions for cross-disciplinary learning but also provide support for cross-context instruction beyond educational coursework.

## Introduction

Educational robots (ERs) have gained popularity in classrooms, as they are considered as effective tools for fostering students’ CT skills. The typical goals of ERs range from a low threshold of generating students’ interest and learning with abstract concepts to a central CT development of problem decomposition, algorithm design, iteration, and debugging (Shute et al., 2017). Many educators have seen their potential and have designed ER activities beyond Science, Technology, Engineering, and Mathematics (STEM) for interdisciplinary activities, such as music (Chung, 2014), arts (Burhans and Dantu, 2017), and foreign language learning (FL; e.g., Hsu and Liang, 2021). According to the review of Papadakis (2021), CT skills can be studied as a problem-solving mechanism and a way that allows users to identify problems or organise the situation by expressing their thoughts, and thus can support cognitive development. Although cross-discipline development was not highlighted in the analysis of Papadakis (2021), being able to express oneself, development of computational fluency, and language development are all essential skills included in CT development (Papadakis, 2021). The current study extrapolated the view of Papadakis (2021) beyond educational coursework on communication development in computational fluency. It aimed to tailor ER activities which integrated CT and target-language (TL) learning, as ERs can be programmed to not only be a medium for CT development, but also to offer unique ways of engaging students in problem-solving tasks while cultivating peer-to-peer communication and interactions in TL learning.

Simply putting these components (ERs, CT, and FL) together, however, does not guarantee the development of the anticipated competences. Indeed, interdisciplinary activities cannot be implemented without carefully designing meaningful ways to develop CT and TL, along with using an appropriate approach. Interdisciplinary activities designed for CT and TL development do not only consider the problem-solving skills involved in coding; they also need to consider allowing students to express themselves and state their thoughts in programming, and to support their TL interaction while lowering their potential TL learning anxiety.

One way to build problem-solving innovators is through establishing collaborative learning settings, as it has been evidenced as an effective approach to help students obtain CT skills while eliciting discussion in programming (Lewis, 2011; Wei et al., 2021). Pair programming (PP) is recommended by researchers as an intervention method that offers collaborative explicit guidelines to instructors on how to integrate ERs and classroom practice (Zhong et al., 2017; Wei et al., 2021). The rationale of PP stems from collaborative learning approaches that require two people to work together and switch roles during the coding process, where one (the driver) operates the device and writes the code while the other (the navigator) offers information by watching for possible defects or directing design decisions. Those who advocate the use of PP argue that it leads to better learning results (e.g., CT), intensive involvement, and communication in coding, and increases students’ satisfaction (e.g., Zhong et al., 2017; Wei et al., 2021). However, results regarding PP are inconsistent, and studies using PP in interdisciplinary activities remain scarce.

Thus, tangible manipulation of ERs was integrated into the PP approach (ER-integrated-PP) to allow children to identify a situation, define a problem, and come up with a solution (Chevalier et al., 2020). Such collaborative activities afford students the opportunity to express their ideas and develop their abilities of problem decomposition, abstraction, algorithm design, debugging, iteration, and generalisation, corresponding to Brennan and Resnick’s framework of the description of computational practice (Brennan and Resnick, 2012; Shute et al., 2017). While engaging in collaboration, the ER-integrated-PP approach appears to be feasible for engaging students in TL interaction and communication in the process of problem-solving learning using target languages.

The premise of acquiring target-language acquisition is a way of activity construction that affords students opportunities for target-language practice (Sato and Storch, 2020). This activity construction also needs to support an open environment that offers students multiple ways to solve a problem in their CT development. A board-game activity adopting a gamified mechanism is a type of communicative task which actively engages students in group talk. The question-and-response interaction tailored in the board-game activity asked students to practise the assigned target sentences, which not only provided opportunities for TL production but also facilitated students’ intensive interaction, echoing interactionist perspectives on second language acquisition (SLA). The board-game activity also supports multiple combinations of CT problem-solving learning (Chen and Chi, 2020). The explicit facilitation of CT learning content embedded in the gamified mechanism can be joined with the promotion of language learning *via* a creative ER-integrated PP approach. Concisely, participants not only carry out the coding development using PP within groups, but also work on the target language and continually interact with each other between groups using the assigned sentence practice in the board-game activity.

When approaching language learners, it is essential to perceive the difference between learning a language in a second language environment (L2) and in a foreign language environment (FL). Past studies have shown that L2 settings are more effective for learning target languages than FL settings (Taguchi, 2008). L2 learners gain more context-rich target language access in natural communication situations compared to EFL learners who cannot access the target language in the immediate environment, although mass media may offer chances for target language practice (Longcope, 2009). While understanding that “contexts matter” significantly affects students’ target-language learning (Sato and Storch, 2020), whether integrating interdisciplinary activities across different contexts shares similar results with a specific subject remains unknown. In addition, a pedagogically-informed instructional design of the learning approach (e.g., PP) and gamified activities (e.g., board-game) can be shared and adapted across contexts. It is pivotal to examine students’ learning performance and to compare their learning behaviours in two distinct contexts; it would then be possible to identify potential challenges and offer insights into curriculum implantation and support for instructors.

While those complex elements are involved in interdisciplinary design to meet anticipated results, reducing potential TL learning anxiety for those who are L2 or FL learners must be taken into account. It was essential to offer a positive learning atmosphere to increase learners’ participation in this cross-discipline study, as these two particular groups across contexts might demonstrate unique learning behaviours, and so diverse and advanced methods were needed to cope with the given task when they first accessed CT concepts while engaging in TL acquisition. Concisely, exploring how these two particular groups of language learners coped with the interdisciplinary learning and potential language-learning anxiety in the proposed approach needed to be highlighted. In this paper, we investigate the impacts of the ER-integrated-PP approach on these two groups of CSL and EFL learners, and examined their learning anxieties and learning behaviours in the proposed approach and activity. Our study results can thus contribute to learning performance in cross-disciplinary development, and provide potential teaching implications and suggestions to support learners in their specific contexts and instructors in their curriculum design.

Using CT skills to deal with coding tasks needs to go through certain phases, while interacting with TL appears challenging for students. It may not be easy to unveil the results without examining students’ behaviours regarding how they conduct ER activities to transform their ideas into problem-solving and solutions while engaging in TL practices. Chevalier et al.’s creative and computational problem solving (CCPS) model (2020) serves as a useful model for evaluating students’ behaviours associated with CT development. It is a comprehensive model that illustrates these key phases: understanding the problem, generating ideas, formulating the robot’s behaviour (the first loop), and programming and evaluating the solution (the second loop). They suggested that practitioners should be aware of ERs’ immediate feedback and of students’ rapid validation of strategies. As ER activities easily drive students into trial-and-error loops, students’ learning without systematic examination of their strategies and justifying their reasons of decomposing problems has often been reported (Shute et al., 2017). Chevalier et al.’s CCPS model (2020) was adapted to help evaluate students’ behaviours and examine the implementation of ER activities associated with CT and target-language development.

However, the above claims require further investigation because the effect of PP on ER-integrated interdisciplinary activities of CT and target-language learning, along with the board-game activity, for young learners is still not well documented. The aim of this study was therefore to design and assess an interdisciplinary activity catering for sixth grade children, and to investigate their learning behaviours when an instructional design took PP and board-game activities into account. An ER-integrated-PP approach with a pedagogy-informed-gamified design was deployed to enhance students’ physical learning experience when compared to learning effects and differences of two groups. The ER-integrated-PP approach with gamified activities may act as a catalyst in promoting embodied learning experience for improving interdisciplinary integration, thereby fostering CT skills and language learning while lowering their anxiety about using the target language from the assigned sentences in this particular setting.

Therefore, the research questions are as follows:

1. Were there any differences in the interdisciplinary learning of the two groups (i.e., CSL and EFL) in the ER-integrated-PP context?
2. Were there any differences in the CT skills of the two groups in the ER-integrated-PP context?
3. Were there any differences in the FL anxiety of the two groups in the ER-integrated-PP context?
4. What were the differences in the learning behaviours of the two groups in the ER-integrated-PP context?

## Literature Review

### ER-Integrated-PP

To deal well with the challenges of the 21st century, attention has been paid to CT skill development. Programming has been a major tool to access CT skills (Hsu et al., 2018) because it allows students to access fundamental skills of abstraction, algorithmic thinking, critical thinking, debugging, and iteration (Shute et al., 2017).

Among the various strategies, PP presents a promising strategy to teach programming. The rationale of PP is that two people, a driver and a navigator, work side-by-side with one computer to generate coding collaboratively, while regularly switching roles. The driver mainly operates the computer to generate code, whereas the navigator contributes ideas and directions for solving problems (Williams and Kessler, 2002). Zhong et al. (2017) provided a clear summary of the benefits of PP, including cognitive development of CT and programming skills, increasing retention and learning satisfaction, and better communication, cooperation, and teamwork. Wei et al. (2021) further evidenced the effect of CT skills in PP while promoting the development of soft-skills (self-efficacy) for young learners. The studies of Zhong et al. (2017) and Wei et al. (2021) showed the similarities of the effectiveness of PP for learning CT skills *via* programming; however, ER activities integrated into PP to enhance CT skills and other disciplines have not been fully explored. As we were interested in exploring TL learning when students were situated in conversation practice using the target language, the experiment aimed to monitor the switching between roles of the driver and navigator within the pair of young students (within-group), and to ensure that they carried out their assigned conversation practice when they worked between groups.

### Target-Language Production Enforcement

Although there has been no direct report on improving TL using ERs with PP, PP has been observed to improve soft skills such as communication skills (D’Angelo and Begel, 2017) and collaboration skills (Lewis, 2011) and to reduce learning frustration and anxiety (Zhong et al., 2017). However, TL acquisition in this cross-discipline study may not be easily attained without reinforcing intensive interaction during TL production, although production practice activities have been regular classwork along with well-designed comprehensive input in modern language classrooms (Sato and Storch, 2020). One gamified activity, the board-game activity, appears promising. Apart from sharing gamified characteristics such as immersion, flow, high motivation, and engagement (e.g., Chen and Chi, 2020; Cheng et al., 2020; Kuo and Hsu, 2020), it offers unique advantages that facilitate learners’ high interactivity opportunities and shared learning between participants (Chen and Chi, 2020). Board-game activities allow learners to employ open-ended, low-floored, shared, cooperative, strategic, and creative thinking to access CT (Chen and Chi, 2020). As participants in gamified activities must clearly comprehend all rules and information, and take turns playing to compete with their counterparts (Kuo and Hsu, 2020), such activities have gained popularity owing to the ease of classroom adaptation and implementation.

It is this specific turn-based nature involved in social interaction that allowed the attention on tailored TL production in the current study. It was not reasonable to expect students to communicate with each other using the target language, since both groups across contexts are FL and L2 learners. Rather, the question-and-response interaction underlying the turn-based nature of the board-game activity, along with the assigned sentences and some relevant vocabulary, was tailored to fortify students’ TL production while they were working on their CT development. A series of turns in a session of the board-game activity could not only potentially accumulate TL practice; students could also develop their self-reflections by revising their previous errors in their upcoming turns (Chen and Chi, 2020). This meets the desired goals of the current study for CT development and TL production.

Although past studies presented empirical evidence of the effectiveness of ERs with board-game learning in terms of supporting learning (Hsu and Liang, 2021), such as self-directed and problem-solving abilities (Cheng et al., 2020), few have attempted to integrate board-games into cross-disciplinary and cross-contextual learning using ERs. The current study aimed to fill this gap by applying a pedagogically informed approach and activity that inculcated ER tools in an interdisciplinary learning scenario.

In addition, it must be clarified that there were two dimensions of strengthened collaborative learning involved in the current study: within-group and between-group design. The ER-integrated-PP approach stresses within-group interaction that involves students in meaning negotiation when they interact with partners to interpret or generate new understandings in the given task. The study of Cheng et al. (2020) showed that interactive skills using ERs improved FL learning (Cheng et al., 2020), while the research of Brennan and Resnick (2012) evidenced that ERs can facilitate students’ abilities of problem solving, critical thinking, and cooperation to keep up with twenty-first century needs. Such strengthened collaborative learning on ERs with PP appear beneficial for fostering peer interaction and engagement in the within-group setting.

On the other hand, the question-and-response routine involved in board-game activities emphasises between-group interactions by strengthening target-language output production practice. While being involved in intense collaboration to complete a coding task *via* PP within groups, high interactivity between groups in target-language practices *via* the board-game activity was tailored to comply with interactionist perspectives in SLA, where explicit facilitation of target forms (e.g., sentences and vocabulary) was reinforced to facilitate oral development *via* communicative tasks (Blyth, 2018). Such reinforced interaction in both modes can also jointly create a positive learning environment, thus potentially reducing students’ learning anxiety in this cross-discipline study. Briefly, ERs implemented with PP along with gamified activities in the within-group and between-group design is in line with the pedagogical support aims for achieving the desired goal.

### Language Learning Anxiety

In language learning contexts, anxiety refers to the “worry and negative emotional reaction aroused when learning or using a second language” (MacIntyre, 1999, p. 27). Cumulative findings have shown that anxiety inhibits learners from participation in oral activities (e.g., Cakici, 2016), and negatively predicts outcomes in the second language (L2; e.g., Saranraj and Meenakshi, 2016). However, analysing the cause of anxiety in class with proper instructional design helps teachers understand students’ problems, and they can then try to enhance students’ learning performance (Hu and Wang, 2014). Assisting students in dealing with the conditional anxieties while making the learning environment less stressful are two key strategies to reduce students’ anxiety (Horwitz et al., 1986).

Saranraj and Meenakshi (2016) stated that learning can be effective if anxiety can be appropriately handled or coped with in L2 or other language learning environments, because many learners experience a certain degree of anxiety during their learning, and use specific strategies to cope with it. Thus, instructors play a key role in reducing students’ anxiety by properly designing activities as well as establishing a welcoming learning atmosphere for classroom activities. In the current study, ERs were a medium for the course activity that helped achieve effectiveness of the learning and the technology use, whereas PP strategies plus board-game activities could be a desirable design to offer a warm classroom setting while reducing students’ learning anxiety.

### Learning Behaviours

Analysing students’ behaviours in ER activities is a springboard to understanding students’ strategy use, and how they develop their cognitive processes associated with CT competences (Tsai et al., 2012). Chevalier et al. (2020) established a CCPS model that allows teachers to validate their instructional interventions, and to effectively facilitate students’ CT development. For example, while instructional methods aim to cultivate students to have productive learning associated with reflection and planning of their strategies, they promote repetitive behaviours in the mechanical operation process, known as the trial-and-error approach (Wing, 2006) to improve skill development. Instructors can thus plan some proper instructional interventions that will assist students in building a well-settled strategy in the classroom practice of ERs (Chevalier et al., 2020).

This study adapted the CCPS model. To fit with the current context, two loops were taken from CCPS while one was expanded. The first loop (within-group) involves students in discussing and negotiating their proposed ideas, and reflection on their problem-solving strategies, including understanding the problem, generating ideas, and formulating the robot’s behaviour (interpreted as negotiation loops). The second loop (within-group) is about programming and evaluating the solution (Chevalier et al., 2020), which limits students’ productive learning (identified as evaluation loops). The extended one is about the target-language interaction loop (between-group), where students are engaged in the assigned conversation practice with team-based learning. These three loops modified by CCPS were used to analyse the students’ learning behaviours and find out their ways of dealing with the problem-solving task in the interdisciplinary learning.

## Research Method

### Participants

A total of 32 Grade 6 students participated in this study, 16 of whom were learning Chinese as a second language (CSL) in Singapore, while 16 were learning English as a Foreign Language (EFL) in Taiwan. None of the students had any previous experience of accessing interdisciplinary activities. Both groups participated in a language classroom with several weeks tailored for interdisciplinary activities. They were all volunteers to participate in the task. Both groups’ language proficiency was considered to be at an elementary level. The research team cooperated with both the CSL teachers in Singapore and the EFL teachers in Taiwan to conduct the study in each of their specific contexts.

### Instructional Design

**Figure 1:**
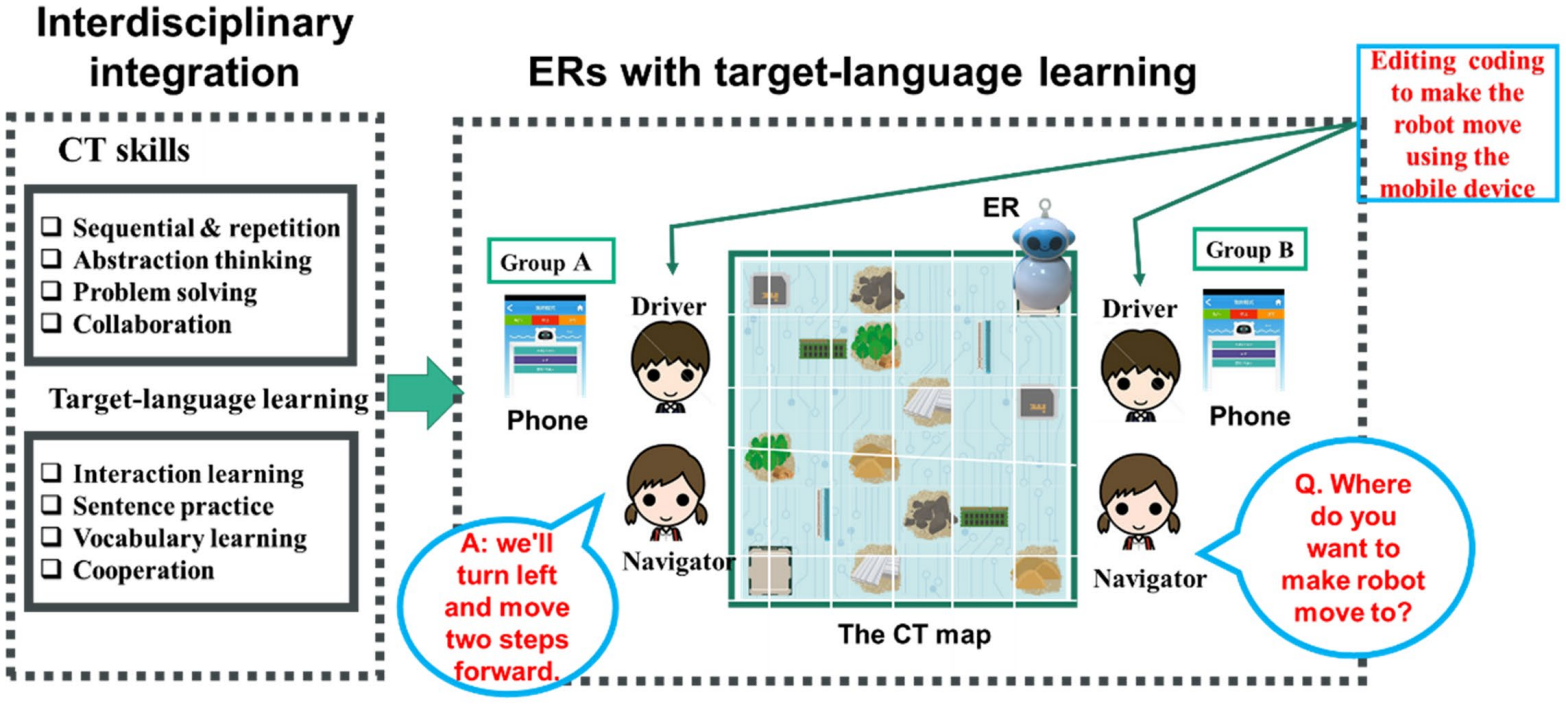
Research framework.

The interdisciplinary activity was tailored to guide students to develop CT skills and target-language development using the interactive ER activity in the language learning classroom (Figure 1). The board-game activity was to collect required materials in the appointed place. While collecting materials, students were involved in developing CT skills by accessing logical sequences, executive conditions, and debugging. After ensuring all the students understood the rules, information, and the ways of controlling and programming the ERs, the CT task was conducted. Meanwhile, relevant vocabulary and sentence patterns for oral interaction were also organised for TL learning, where both groups had exactly the same content (conversation practice) but with different TL and programming interfaces (Chinese vs. English). Students worked in pairs to complete the coding task, and completed the set question-and-response interaction jointly.

**Figure 2:**
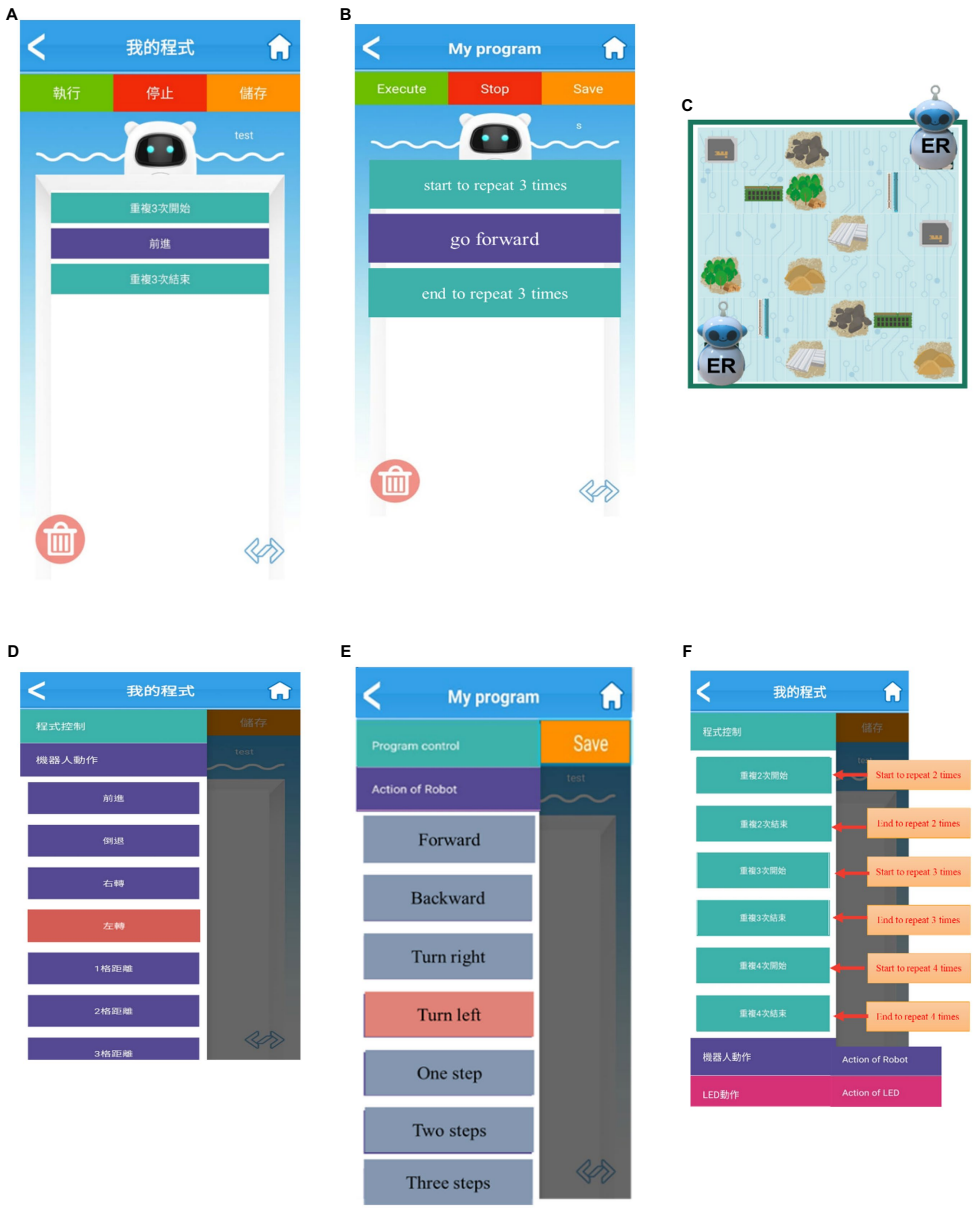
**(A)** ER tools for EFL. **(B)** ER tools for CSL. **(C)** Interactive activity. **(D)** CT learning for EFL. **(E)** CT learning for CSL. **(F)** CT learning.

Briefly, three constructs were established, namely ER tools with block-based programming (Figures 2A,B), CT learning (Figures 2D–F), and target-language materials (Table 1). The first are the ER tools. The study used physical motor-based ERs, where motors, sensors, and memory, and a map with infrared-reflection and identifiers were all included to control the ERs on the map (Figure 2C). This allowed students to simultaneously test their coding.

**Table 1:** Example of target-language learning materials for both groups.

When the explorer of the opposite team asked the question, say: *Where do you want to make the robot move to? 你想要機器人往哪裡走?* The explorer of the team answered the question, saying: *We want to move one step forward. 我們想前進一步* *We want to move two steps forward and turn right/left. 我們想前進二步，然後右/左轉* *We want to turn right/left and move three steps forward.我們想先右/左轉,然後前進三步*

Second is CT learning. As the scenario of the board-game interactive activity asked students to construct city buildings by collecting needed materials (e.g., stones), students needed to identify their targets, decompose how the ERs could reach their intended destinations (e.g., wood), and come up with solutions (using algorithms like sequential logic or loop) or debugging (Figure 2F). They worked in pairs to control the robots by operating the block-based app programming to research the targeted place or to obtain the needed resources. Two teams worked at a table and competed with each other. CT learning occurred when the pairs of students controlled the ERs by moving them in the anticipated way.

The third construct was to arrange relevant vocabulary and simple sentence practice while students were working on their CT learning (see an example in Table 1). Students needed to apply these words and sentences in the CT activity. The vocabulary included words and phrases such as “move, step, forward and move, turn right or left, sand, construction, and stone.” Two teams were involved in practising the conversations with each other using question-and-response exercises in the turn-based board-game activity.

**Figure 3:**
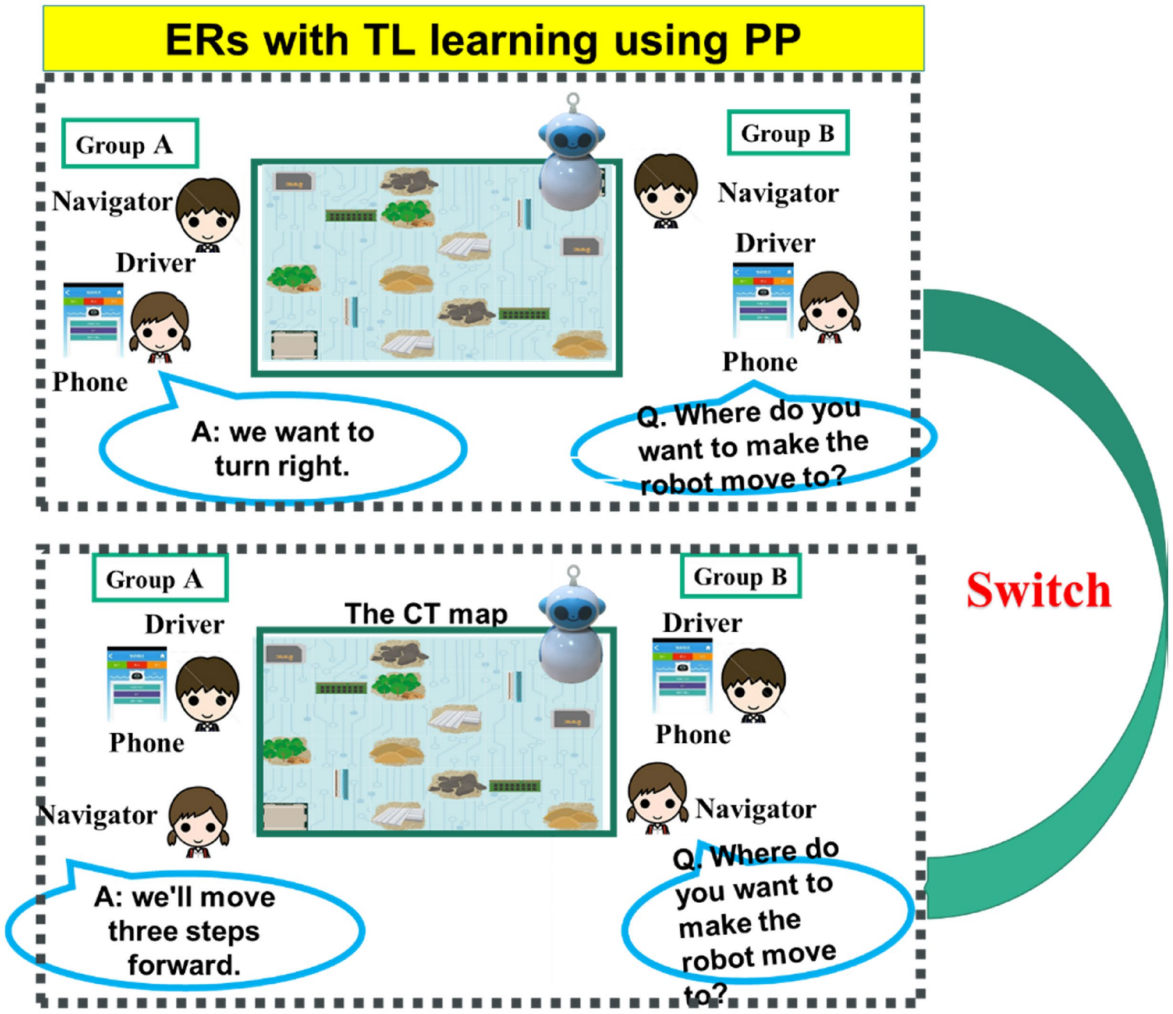
The implementation of the ER-integrated-PP approach along with the question-and-response interaction for both groups.

Both groups accessed the same materials, including basic sequential rules and the algorithm of simplifying the steps, and vocabulary and sentence pattern teaching. They also received similar learning instruction and strategies (Figure 3), emphasising the roles of navigator and explorer, and TL conversation practice. After the explorer of one team operated the app to control the ERs, the navigator needed to ask the navigator of the opposite team to answer the questions in the target language (i.e., English or Mandarin). The navigator of the opposite team answered the question after completing coding. The two teams switched with their own partners to engage in TL practice. Shortly, the ER-integrated PP approach emphasised the two roles of explorer and navigator within teams (within-group interaction), while question-and-response interaction in the board-game activity involved language practice between groups (between-group interaction) in the interactive ER activity.

### Research Process

A quasi-experimental method was adopted in this study, including a 3-week experiment period with one session of language learning for two weekly lectures of 1 h, and the other session of CT integration for one weekly activity of 2 h, giving a total of 4 h per week.

In the previous two periods, the students took the pre-test of language learning (either Mandarin or English) and completed the pre-questionnaire of the computational thinking scales (CTS) and the language classroom anxiety scale. They learnt the vocabulary and conversation practice in English for the EG and Mandarin, and received mini-guidance on the basic CT concepts. The last period was arranged to implement a hands-on activity in which students applied the learnt CT concepts and the relevant conversation practice involved in the interactive ER activities. Then, the post-test of English and Mandarin, and the post-questionnaires of CTS and language-learning anxiety were administered in the language classroom. Lastly, the learning process was video-recorded; their behaviours were further analysed for later discussion.

### Instruments

The pre-test and post-test of CT competencies (50%) and language proficiency (50%) comprised: (1) sentence combination (five items worth 10 points) and multiple-choice questions for vocabulary items (10 items worth 40 points), with a full score of 50 for CT competences, and (2) the same arrangement of tests for language proficiency, with a full score of 50 for language proficiency. The test items were consistent with the goal, where students used TL in their participation in the question-and-response interaction. Both tests (Mandarin or English) had the same test content, but items were written in the different target languages (see Appendix). One experienced English teacher, one Mandarin teacher, and one technology education teacher were invited to validate both tests with the two different target languages. The researchers along with the two experts ensured the validity of the tests.

The questionnaire of CTS, adapted from the computational thinking scales by Korkmaz et al. (2017), with a 5-point Likert scale (1 = *strongly disagree*; 5 = *strongly agree*) was adopted to evaluate the students’ knowledge, skills, and attitudes towards creativity, algorithmic thinking, cooperativity, critical thinking, and problem solving. This study adapted four dimensions of six items for the algorithmic-thinking dimension, four items for the operation dimension, five items for the critical-thinking dimension, and six items for the problem-solving dimension, with a Cronbach’s alpha value of 0.82, showing acceptable reliability.

The language-learning anxiety scale was employed from Horwitz’s FLCAS questionnaire (Horwitz, 1986), with a 5-point Likert scale (1 = *strongly disagree*; 5 = *strongly agree*). FLCAS was also modified for the CSL learners to examine their learning anxiety. This study used 10 items for speech anxiety, four for communication apprehension, three for negative evaluation, two for fear of making mistakes in target-language class, and nine for feeling uniquely unable to deal with the task of L2, with a Cronbach’s alpha value of 0.78, showing acceptable reliability.

### Coding Scheme

**Figure 4:**
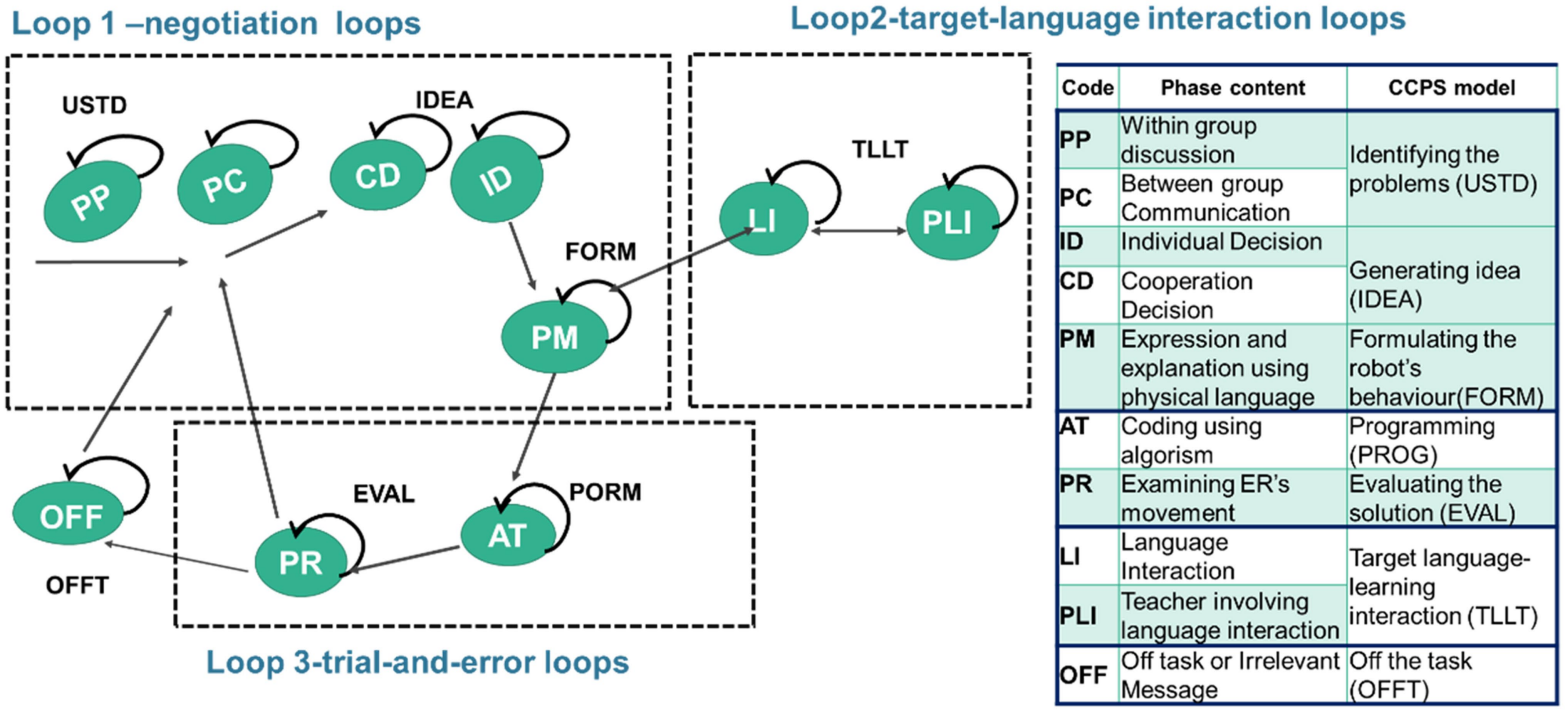
The model for evaluating students’ learning behaviours.

The study investigated students’ learning behaviours regarding the CT and TL learning in the ER-integrated interdisciplinary task. Students’ behaviours involved in tasks between and within teams were captured, and every 10s was a note to locate the main action in line with the video coding technique. To clearly identify students’ behaviours during activities, two experts experienced in CT and target-language integration (FL and L2) were invited to confirm the coding schemes associated with the CCPS model to ensure the reliability of the behavioural analysis in this study. These experts together with the researchers confirmed the suitability of the codes and the corresponding CCPS definitions. Based on actual action on CT or FL interaction recorded in the video, the behaviours under the categories pertaining to CCPS were finally identified in the coding scheme listed in Appendix 1 (Figure 4).

## ResultS

### Learning Achievement

The purpose of this study was to examine if CSL and EFL had different learning outcomes when students were taking part in the interdisciplinary activities of language and CT integration. A significant difference was observed from the *t*-test results of the pre-test scores of the two groups (*t* = −4.991, *p* > 0.05), meaning that the homogeneous hypothesis of the two groups’ achievements before the activity was violated. This implied that directly investigating the progress effects of dependent variables was reasonable. The result showed that no significant difference was found for language-learning progress in the independent sample *t* tests (*t* = 0.23; *p* = 0.812 > 0.05) between CSL (*M* = 10.00) and EFL (*M* = 9.13). However, a significant effect was observed for CT progress (*t* = 3.02; *p* = 0.005 < 0.05) and post-test progress (*t* = 0.81; *p* = 0.009 < 0.05). The CSL group had significantly higher progress performance in CT progress (*M* = 19.75) and post-test progress (*M* = 29.75) in comparison with the EFL group in CT progress (*M* = 5.63) and post-test progress (*M* = 14.75), when participating in this learning activity (Table 2).

**Table 2:** Progress scores of the independent sample *t*-test results between the two groups.

	CSL			EFL			*t*	*p*
	*N*	Mean	*SD*	*N*	Mean	*SD*		
Language progress	16	10.00	13.19	16	9.13	6 30	0.23	0.812
CT progress	16	19.75	17.71	16	5.63	5.88	3.02^**^	0.005
Total progress of learning achievement	16	29.75	20.20	16	14.75	7.52	2.81^**^	0.009

** *p* < 0.01.

**Figure 5:**
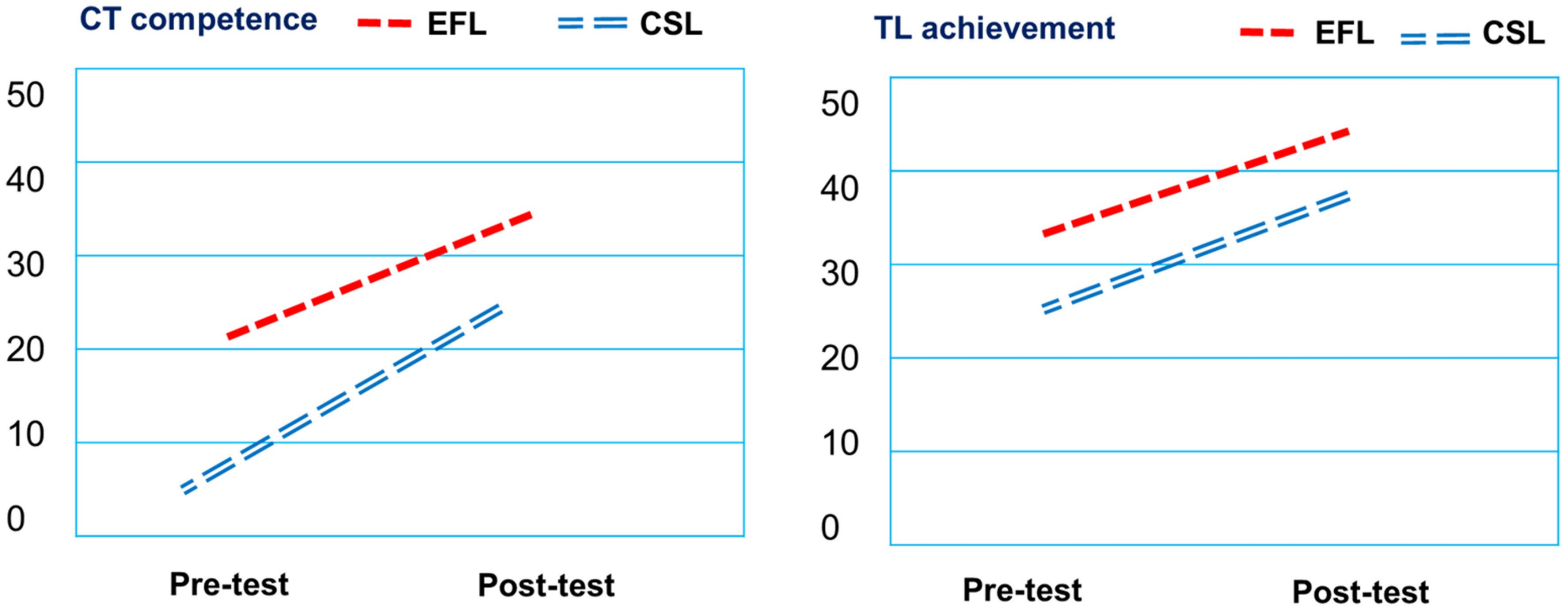
Pre-test and post-test of CT and TL learning scores of the EFL and CSL students.

Further, paired sample *t* tests were used to investigate the progress of both groups. Both groups significantly improved in their language learning (*t* = −3.03^*^; *p* < 0.05 for CSL, and t = −5.79^***^; *p* < 0.05 for EFL), CT capacity (*t* = −4.46^***^; *p* < 0.05 for CSL and *t* = −3.83^**^; *p* < 0.05 for EFL), and overall learning achievement (*t* = −5.94^***^; *p* < 0.05 for CSL and *t* = −7.84^***^; *p* < 0.05 for EFL). Both groups made significant improvement in their linguistic knowledge of target language process, CT process, and overall process of learning achievement, showing that the interdisciplinary activities were beneficial for integration acquisition (Figure 5).

### Computational Thinking

The study aimed to examine the effects of the different learning groups on students’ CT. One-way ANCOVA was first conducted using the pre-questionnaire scores of CT as a covariate. After verifying that the assumption of homogeneity of regression was not violated with *F* = 1.24 (*p* > 0.05), the post-questionnaire scores of the two groups were analysed. However, no significant effect was found between independent variables (*F* = 0.247, *p* > 0.05) on the students’ CT skills (Table 3).

**Table 3:** Descriptive statistics of the two groups.

	CSL group (*N* = 16)					EFL group (*N* = 16)				
	Pre-test		Post-test			Pre-test		Post-test		
	*M*	*SD*	*M*	*SD*	*AdjM*	*M*	*SD*	*M*	*SD*	*AdjM*
Algorithm	2.98	1.02	3.27	0.82	3.27	3.28	1.13	3.30	1.02	3.30
Cooperation	3.57	0.90	3.30	0.68	3.30	4.05	0.98	4.03	0.92	4.03
Critical thinking	3.35	0.93	3.05	0.91	3.05	3.33	0.97	3.44	0.82	3.44
Problem-solving	2.96	0.72	3.38	0.76	3.38	2.17	1.13	2.47	1.03	2.47

MANCOVA was further conducted using the pre-questionnaire scores of CT as a covariate to eliminate the learners’ differences in their equivalent prior knowledge before the task, after verifying that the assumption of homogeneity of regression was not violated with *F* = 0.913 (*p* > 0.05) and that the Box’s M test for homogeneity of covariance matrices was not violated with (Box’s *M* = 21.35, *F* = 1.82, *p* > 0.05).

Table 4 shows that the four subscales of CT in the post-questionnaire differed significantly between the two groups (Wilks’ lambda = 0.58, *F* = 4.62, *p* = 0.006, Eta = 0.42). The Bonferroni method was then used to analyse the confidence intervals. The results of the *post hoc* comparison indicated that the EFL group showed better cooperation than the CSL group, while the CSL group showed greater problem-solving capacities than the EFL group in these four dimensions of CT regarding algorithm, cooperation, critical thinking, and problem-solving skills.

**Table 4:** *MANCOVA* analysis of CT for both groups.

*SV*	*Df*	*SSCP*				Wilks’ lambda	*F*			
	1	Algor.	Coop.	Critical	Prob-solv		Algor.	Coop.	Critical	Prob-solv
Between Group	1	0.242 1.069 0.706 −1.226	1.069 4.722 3.119 −5.418	0.706 3.119 2.060 −3.579	−1.226 −5.418 −3.579 6.217	0.584^**^	0.268^NS^	6.176^*^ (E > C)	2.677^NS^	8.0444^**^ (C > E)
Pre-test	1	0.695 0.283 0.745 1.398	0.283 0.115 0.304 0.569	0.745 0.304 0.799 1.498	1.398 0.569 1.498 2.810	0.872	0.771	0.175	1.038	3.636
Within Group (error)	29	26.138 13.848 19.418 2.556	13.848 19.084 13.893 −2.097	19.418 13.893 22.316 6.692	2.556 −2.097 6.692 22.415					
Sum	32									

E, ESL; C, CSL; Algor., algorithm; Coop., cooperation; Critical, critical thinking; Prob-solv, problem-solving skills.

** *p* < 0.01;

* *p* < 0.05;

NS *p* > 0.05.

### Learning Anxiety

The study aimed to examine the effects of the different learning groups on students’ anxiety. One-way MANCOVA was conducted using the pre-questionnaire scores of learning anxiety as a covariate, after verifying that the assumption of homogeneity of regression was not violated with *F* = 1.939 (*p* > 0.05) and that the Box’s M test for homogeneity of covariance matrices was not violated with (Box’s *M* = 24.93, *F* = 0.158, *p* > 0.05).

Table 5 presents that the five subscales of learning anxiety in the post-questionnaire differed significantly between the two groups (Wilks’ lambda = 0.408, *F* = 7.26, *p* < 0.001, Eta = 0.59). The Bonferroni method was then used to analyse the confidence intervals. The results of the *post hoc* comparison indicated that the EFL group showed lower learning anxiety than the CSL group for the dimensions of speech anxiety, communication apprehension, and fear of being negatively evaluated by other students.

**Table 5:** *MANCOVA* analysis of learning anxiety for both groups.

*SV*	*Df*	*SSCP*					Wilks’ lambda		*F*			
	1	SA	*CA.*	FN	FM	UT		SA	*CA.*	FN	AM	FU
Between Group	1	14.92 11.50 11.06 9.65 5.37	11.502 8.867 8.528 7.439 4.145	11.063 8.528 8.203 7.155 3.987	9.650 7.439 7.155 6.241 3.478	5.377 4.145 3.987 3.478 1.938	0.408^**^ ^*^	27.56^**^ ^*^ (E < C)	14.03^**^ (E < C)	12.36^**^ (E < C)	5.15^*^ (E < C)	3.26^NS^
Pre-test	1	0.228 0.165 −0.195 0.165 0.331	0.165 0.119 −0.141 0.119 0.239	−0.195 −0.141 0.167 −0.142 −0.283	0.165 0.119 −0.142 0.120 0.240	0.331 0.239 −0.283 0.240 0.480	0.862	0.421	0.161	0.252	0.099	0.806
Within Group (error)	29	15.70 15.79 14.50 16.52 12.31	15.79 21.38 16.93 22.41 13.57	14.50 16.93 19.23 17.34 11.55	16.52 22.41 17.34 35.13 14.09	12.31 13.57 11.55 14.09 17.24						
	31											

E, ESL; C, CSL; SA, speech anxiety; CA, communication apprehension; FN, Fear of being negatively evaluated. AM: fear of making mistakes in class; FU, Feeling uniquely unable to deal with the task.

*** *p* < 0.001;

** *p* < 0.01;

* *p* < 0.05;

NS *p* > 0.05.

### Learning Behaviours

**Figure 6:**
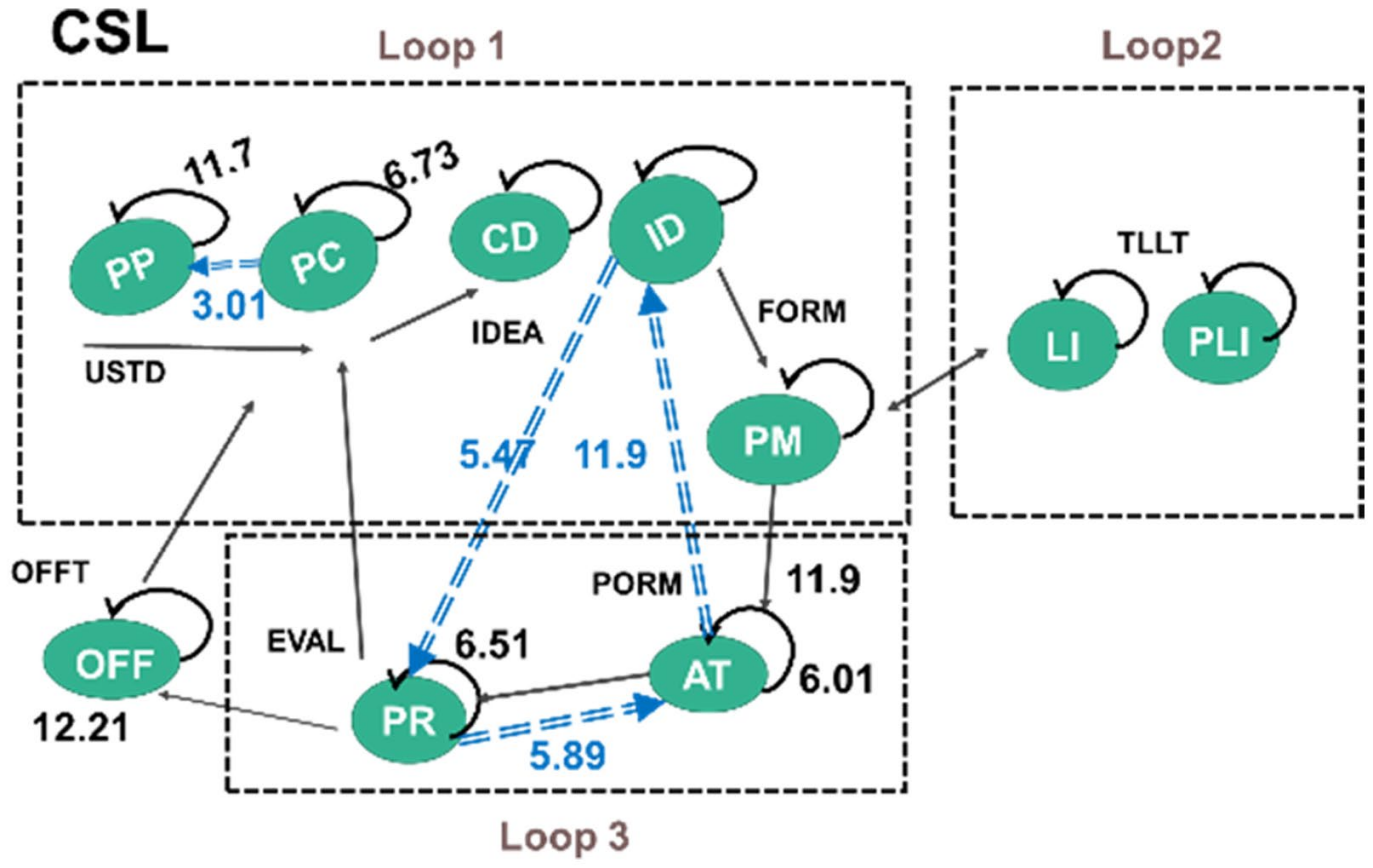
CSL’s behaviour patterns.

**Figure 7:**
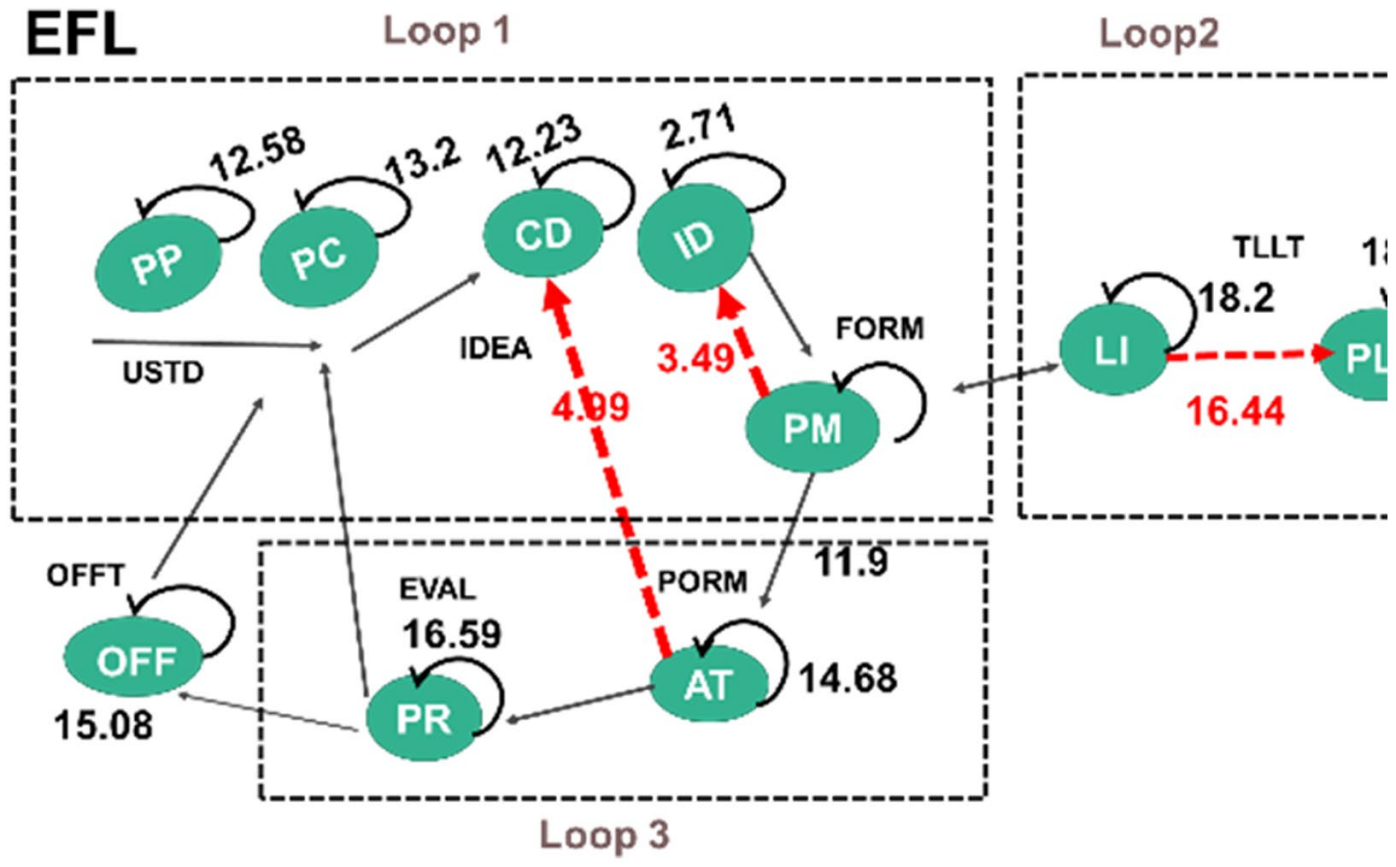
EFL’s behaviour patterns.

In answering the fourth research question, sequential behaviour analysis was executed to examine the differences between the learning behaviours of the two groups. The behavioural sequence reaches a significant level (*p* < 0.05) when the *Z* value is more than 1.96 (*Z* > 1.96; Bakeman and Gottman, 1997). Figures 6 and 7 present the behavioural transition diagrams of the students involved in two learning groups; the *z*-scores are shown on the middle line and each line’s direction represents its transfer direction. Three loops were analysed based on the analysis of Chevalier et al. (2020) of the CCPS model (Loop 1 and 3) and the loop for FL interaction (Loop 2).

**Figure 8:**
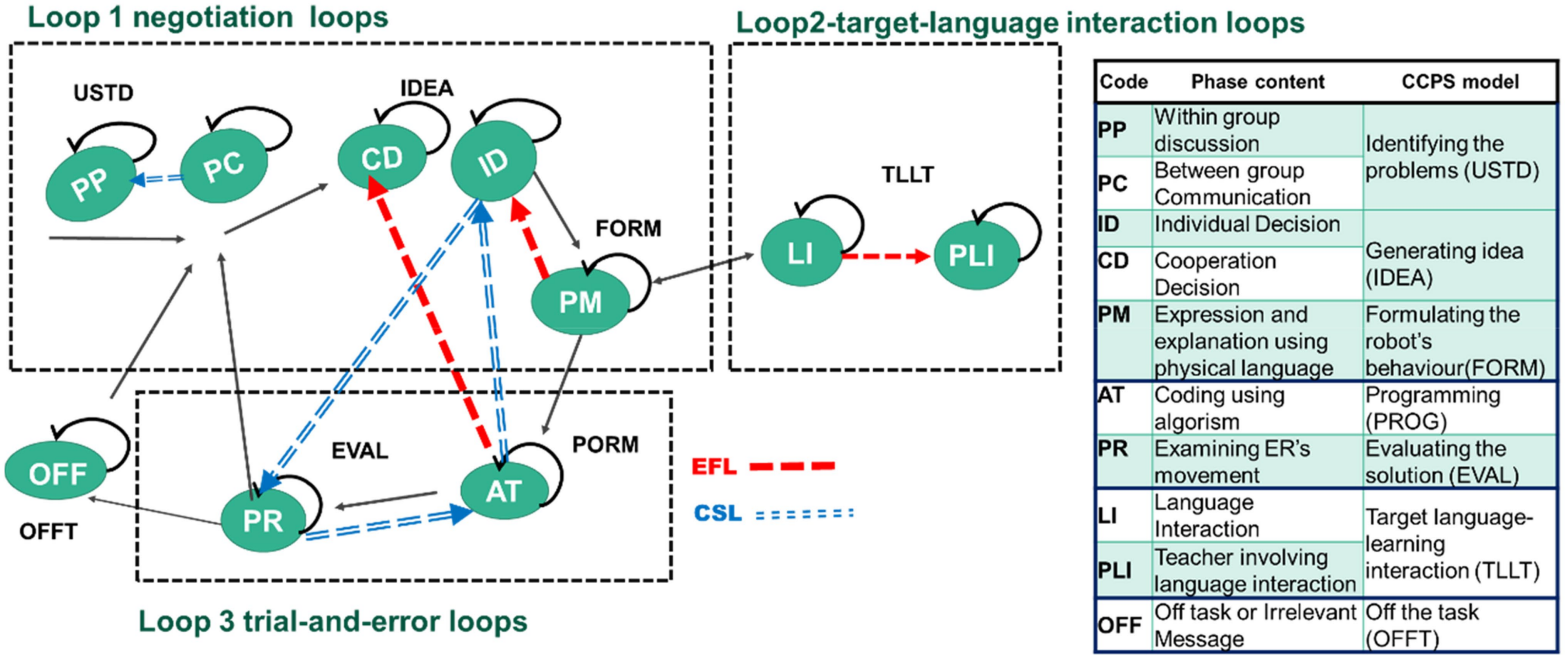
The differences in the two groups’ behaviour patterns.

The main differences between the two groups are that the CSL students presented two statistically significant behaviour sequences involved in loops 1 and 3, clarified as the negotiation loop and the trial-and-error loop, without loop 2, identified as target-language interaction. In comparison, the EFL students demonstrated three statistically significant behaviour sequences in these three loops (see Figure 8).

The CSL’s two significant behaviour sequences are: PC → PP, and ID → PR → AT → ID. During the activities, the CSL students (freely switching roles in the task) demonstrated their behaviours of first identifying the question within or between groups (PC → PP). They then devoted themselves to working on the trial-and-error loop (loop 2), where they worked individually to decide the ER’s routes and started checking the movement of robots to figure out the algorithm to make the ER reach the intended destination (ID → PR → AT → ID). Without focusing the negotiation on solutions and problem-solving strategies as was expected to be seen in Loop 1, the CSL students revealed no significant behaviours of target-language interaction (Loop 2). Students rarely participated in assigned target-language use and interaction.

Otherwise, the EFL’s three significant behaviour sequences are: AT → CD, PM → ID, and LI → PLI. When aiming to reach the intended destination they demonstrated Loops 1 and 3. The EFL students collaboratively generated ideas by working on algorithms (AT → CD), and they physically expressed their ideas using gestures individually to justify their CT concepts to their partners (PM → ID). Such formulation fell into the essence of negotiation on problem-solving strategies, and thus the EFL students revealed their behaviours of significantly engaging in target-language interaction in Loop 2 (LI → PLI). Following the PP task of coding and conversation practice, the EFL students frequently interacted with one another, kept concentrating on the robots’ movements, and used assigned English sentences when it was their turn. If errors occurred, the teacher would come by and guide them to use the taught sentence in their interaction (LI → PLI).

## Discussion

Using ERs to acquire CT has led educators internationally to integrate CT into cross-content areas and to further go beyond STEM into other formal curricula (Cheng et al., 2020; Hsu and Liang, 2021). Understanding that this idea needs to be supported by meaningful task design and pedagogically-informed approaches, ER activities to enhance CT and TL must not solely focus on problem-solving skills. Rather, they must provide students with interactive learning settings to engage them in the cross-disciplinary learning, supporting their cognitive (CT) and TL development while reducing their TL anxiety. Coupled with existing studies that have successfully demonstrated learning outcomes across subjects using ERs (e.g., Hsu and Liang, 2021), critically designing activities with instructional strategies and examining their effects across contexts are scarce. Future research needs to consider whether any available tools (e.g., ERs) with strategies and approaches are meaningful, suitable, and engaging for the particular context in which students are to engage in cross-disciplinary study, as simply putting relevant components together cannot guarantee the anticipated results.

Learning *via* a pedagogy-informed approach and tailored ER activities, both groups made significant improvement in their learning of CT and TL. We make no claims about which particular group is superior to the other when looking into differences in the learning behaviours of the two groups. However, the findings demonstrated in this study are worth considering as pioneering and challenging for cross-context instructors and CT practitioners, who need to be mindful of what requirements and expectations of interdisciplinary activities are deployed to children when they learn to code while accessing TL in coding tasks. Indeed, the results showed that the integration of essential CT skills and TL output production in within-group and between-group interactions could be jointly developed using PP strategies and board-game activities as proposed in this study. The ER-integrated activity, along with PP and board-game activities, can be developed using an interactive design offering an open environment for multiple problem-solving solutions but acceptable challenge for TL output practice. This design is regarded as important as studies investigating cross-disciplinary learning from educational settings argue that if children do not find coding tasks engaging, they will not be involved in discussion and negotiation with others regarding any potential CT strategies (Chevalier et al., 2020), nor will they interact with their counterparts while playing the board-game (Hsu and Liang, 2021).

While pedagogic approaches (e.g., PP) and gamified activities (board-game) are often shared across contexts, critically analysing how they affect students’ learning is often limited to local contexts. Comparing students’ learning behaviours in these two distinct contexts (L2 and FL) reveals potential challenges in interdisciplinary learning across contexts. The CSL students who had natural communication situations favoured individual working and decision making, whereas the EFL students who did not have natural communication situations preferred meaning negotiation and enjoyed collaboration with their partners. The CSL students showed more confidence in facing the challenge of the task by breaking the rules to work independently on solving the problems. However, analysis of their learning behaviours presented a typical trial-and-error loop, although there was a significant difference in their CT progress and problem-solving ability.

A trial-and-error loop is a universal strategy, particularly for novices, for building learning and action in enhancement learning (Mohr et al., 2018); it quickly supports progress (Sutton and Barto, 2018), but not skills development in the long run (Chevalier et al., 2020). In our study, the L2 students’ (CSL) behaviours corresponded to the findings of Chevalier et al. (2020) about novices’ ERs use for CT development. While ERs offer prompt feedback without intervention in the design, and students easily receive immediate evaluation of their strategy, they can fall into a trial-and-error loop. Although it could be possible that the CSL students were fully attracted by the ERs, such quick feedback from the ERs “reduces the potential of learning how to code to a problem, ignoring the expressiveness and communicative functions of programming” (Bers, 2020, p. 503).

While facing this challenge, Chevalier et al. (2020) successfully adopted an intervention of pause strategies in the ER interface to stop students from directly executing the code. Students showed better interaction and strategies as a result. Although their foci were neither interdisciplinary integration nor cross-context investigation, proper intervention to activate students’ communication during programming should be reconsidered for CSL students in the design. If their interaction is activated, CSL students may possibly demonstrate less anxiety in their TL production, since individual work with little attention on between-group interaction often fails to develop social skills and interpersonal relationships, and negatively harms TL acquisition. Although factors affecting students’ language gains are rather complex, it is difficult for L2 students to acquire TL, even though the learners gain much exposure to the target language outside of class (e.g., Taguchi, 2008).

Rather than having a natural communication setting, the EFL students accessed the TL in the formal language classroom. During the task, they showed clear negotiation within groups and TL interaction loops between groups, favouring cooperative decisions. This finding echoes previous studies in EFL settings which found that the language development of learners in a formal FL classroom was facilitated, as they gained abundant input and language use opportunities over time with the help of instructors’ explicit instruction and in-class collaborative peer activities (e.g., Taguchi, 2008; Sato and Storch, 2020). Such interaction can be attributed to the fact that they enjoyed the problem-solving task using a communicative task, since their social skills of cooperation in CTS and anxieties in TL anxiety were significantly better and lower, respectively, than those of CSL students.

PP along with the board-game activity, with an emphasis on switching roles in the coding task and the turn-based nature of playing, also contributed to anticipated loops for the EFL students, where they shifted their attention from using different strategies to negotiate with their partners, reinterpret their ideas, and practise conversational sentences within and between groups. PP interaction echoes the discussion of Zhong et al. (2017), and it was found that switching roles between drivers and navigators within groups enhanced negotiation. They suggested that negotiation is more crucial than switching because it is the essence of collaborative learning, although they neither used ERs nor included interdisciplinary activities in their study. The turn-based nature embedded in board-game activities is evidenced to afford creating a question-and-response interaction to TL output productions. Indeed, switching roles along with turn-based interaction can be considered optimal, as it also supports self-reflection and students learn from their mistakes (Chen and Chi, 2020; Wei et al., 2021).

While students enjoyed collaboration with their partners, they demonstrated lower anxiety, as shown by the EFL students. They had lower anxiety associated with CT development, including speech anxiety, communication apprehension, fear of being negatively evaluated, and fear of making mistakes in the class. Such lower anxiety is important for students’ continued motivation and willingness to use the FL during the coding task. This confirms the finding of Dewaele and Pavelescu (2021) that less anxious learners are often associated with positive experience when trying to comprehend or speak the target language.

Lastly, several limitations should be clarified. One possible factor affecting the research findings is the novelty of the ERs, although both groups did improve their learning achievement and increased their CT skills. Exploring participants’ learning effects in ER-integrated-PP strategies in the long run needs to be addressed. Second, the interactive coding task associated with the board-game mechanism was not described in detail in this paper due to the limited word count. Working on CT integration is not a privilege reserved only for the ER-integrated PP approach. The gamified mechanisms (e.g., level, credits, strategies, competition, and self-reflection) could have been influential variables that came into play to generate the outcomes. If the mechanisms are not appropriate and feasible with regard to the objectives, students may not find the learning task interesting, nor will they engage in it to gain any possible educational benefits (Hsu and Liang, 2021). Future studies addressing board game mechanisms integrated into ER using PP are recommended. Meanwhile, it is hard to generalise the result as the small sample size included in this study. However, the results from the current study serve as pioneering for cross-context instructors and CT practitioners. Lastly, although ER-integrated-PP methods support instructors in integrating interdisciplinary learning activities, it is necessary to confirm the findings from other settings or cross-subject integration.

## Conclusion

A limited number of studies have focused on ER for interdisciplinary activities. Concerning the fact that PP along with board-game activities is one of the great instructional strategies, ER-integrated-PP activities for interdisciplinary activities which integrate CT and TL should not be ignored. This study investigated the feasibility of using ER-integrated-PP activities and critically validated its impact on interdisciplinary learning in the elementary setting. The result concluded that the ER-integrated-PP approach plus the board-game activity for the promotion of interdisciplinary learning was helpful for promoting the two groups of students’ learning in terms of their CT competencies, TL learning, and CT skills, as well as lowering their FL learning anxiety. The results help expand the literature on the design of ERs in a PP way with the gamified activity for interdisciplinary activities.

Students’ learning behaviours revealed that ER-integrated-PP education is adventurous in involving students in developing the CT process related to the essence of pair negotiation on problem identification and reinterpretation, and target-language interaction on the gamified mechanism. Drawing on the findings described above, ER activities for interdisciplinary integration can be feasible at the elementary education level. Researchers interested in ER-integrated-PP associated with board-game activities for interdisciplinary learning could consider if the same cross-context design can be reproduced in other cross-subject areas or institutions.

## Data Availability Statement

The original contributions presented in the study are included in the article/Supplementary Material, further inquiries can be directed to the corresponding author.

## Author Contributions

T-CH: conceptualization, investigation, data curation, writing—original draft, and writing—review and editing. CC: formal analysis, methodology, and writing—review and editing. L-KW and C-KL: formal analysis, data curation, and writing—review and editing. All authors contributed to the article and approved the submitted version.

## Funding

This study is supported partly by the Ministry of Science and Technology of China under contract number MOST 108-2511-H-003-056-MY3.

## Conflict of Interest

The authors declare that the research was conducted in the absence of any commercial or financial relationships that could be construed as a potential conflict of interest.

## Publisher’s Note

All claims expressed in this article are solely those of the authors and do not necessarily represent those of their affiliated organizations, or those of the publisher, the editors and the reviewers. Any product that may be evaluated in this article, or claim that may be made by its manufacturer, is not guaranteed or endorsed by the publisher.
