# Corrigendum: Effects of a pair programming educational robot-based approach on students' interdisciplinary learning of computational thinking and language learning

**DOI:** 10.3389/fpsyg.2022.1054561

**Published:** 2022-11-02

**Authors:** Ting-Chia Hsu, Ching Chang, Long-Kai Wu, Chee-Kit Looi

**Affiliations:** ^1^Department of Technology Application and Human Resource Development, National Taiwan Normal University, Taipei City, Taiwan; ^2^Faculty of Artificial Intelligence in Education, Central China Normal University, Wuhan, China; ^3^National Institute of Education, Nanyang Technological University, Singapore, Singapore

**Keywords:** interdisciplinary activities, educational robots, pair programming, language learning, trial-and-error loops

In the published article, there was an error in Table 2 as published. The *N* value in the EFL column was stated as 15 but should be 16. The corrected Table 2 appears below.

**Table 2:** Progress scores of the independent sample *t*-test results between the two groups.

	**CSL**			**EFL**			***t***	***p***
	***N***	**Mean**	***SD***	***N***	**Mean**	***SD***		
Language progress	16	10.00	13.19	16	9.13	6.30	0.23	0.812
CT progress	16	19.75	17.71	16	5.63	5.88	3.02^**^	0.005
Total progress of learning achievement	16	29.75	20.20	16	14.75	7.52	2.81^**^	0.009

** *p* < 0.01.

In the published article, there was also an error in the abstract as published. The number of participants in each of the Grade 6 classes was stated as 15 but should be 16. The corrected abstract appears below.

Using educational robots (ERs) to integrate computational thinking (CT) with cross-disciplinary content has gone beyond Science, Technology, Engineering, and Mathematics (STEM), to include foreign-language learning (FL) and further cross-context target-language (TL) acquisition. Such integration must not solely emphasise CT problem-solving skills. Rather, it must provide students with interactive learning to support their target-language (TL) interaction while reducing potential TL anxiety. This study aimed to validate the effects of the proposed method of pair programming (PP) along with question-and-response interaction in a board-game activity on young learners' CT skills and TL learning across contexts. Two Grade 6 classes, one with 16 students who were studying Chinese as a Second Language (CSL) and the other with 16 students who were studying English as a Foreign Language (EFL), participated in the activity. A series of instruments on achievement assessment, questionnaires on CT skills and TL anxiety, and sequential learning behaviour analysis were used to critically examine the results. The main conclusion is that the EFL group showed better social skills of cooperation on CT and lower TL learning anxiety, while the CSL group demonstrated better problem-solving skills in CT, but presented more behaviours of trial-and-error loops. Results not only contribute suggestions for cross-disciplinary learning but also provide support for cross-context instruction beyond educational coursework.

In the published article, there was also an error in Research Method, Participants, Paragraph 1. The number of participants was wrongly stated as 30 but should be 32. Also the number of participants learning Chinese as a second language (CSL) and learning English as a Foreign Language (EFL) was stated as 15 when it should be 16. The corrected paragraph appears below:

A total of 32 Grade 6 students participated in this study, 16 of whom were learning Chinese as a second language (CSL) in Singapore, while 16 were learning English as a Foreign Language (EFL) in Taiwan. None of the students had any previous experience of accessing interdisciplinary activities. Both groups participated in a language classroom with several weeks tailored for interdisciplinary activities. They were all volunteers to participate in the task. Both groups' language proficiency was considered to be at an elementary level. The research team cooperated with both the CSL teachers in Singapore and the EFL teachers in Taiwan to conduct the study in each of their specific contexts.

In the published article, there was also an error in Results, Learning achievement, Paragraph 1. In sentence 2, the *p*-value was given as “*p* = 0.000 > 0.05” but should be “*p* > 0.05.” The corrected paragraph appears below.

The purpose of this study was to examine if CSL and EFL had different learning outcomes when students were taking part in the interdisciplinary activities of language and CT integration. A significant difference was observed from the t-test results of the pre-test scores of the two groups (*t* = −4.991, *p* > 0.05), meaning that the homogeneous hypothesis of the two groups' achievements before the activity was violated. This implied that directly investigating the progress effects of dependent variables was reasonable. The result showed that no significant difference was found for language-learning progress in the independent sample *t* tests (*t* = 0.23; *p* = 0.812 > 0.05) between CSL (*M* = 10.00) and EFL (*M* = 9.13). However, a significant effect was observed for CT progress (*t* = 3.02; *p* = 0.005 < 0.05) and post-test progress (*t* = 0.81; *p* = 0.009 < 0.05). The CSL group had significantly higher progress performance in CT progress (*M* = 19.75) and post-test progress (*M* = 29.75) in comparison with the EFL group in CT progress (*M* = 5.63) and post-test progress (*M* = 14.75), when participating in this learning activity (Table 2).

In addition, there was also an error in Results, Learning behaviours, Paragraph 1. The paragraph previously stated, “Three loops were analysed based on the analysis of Chevalier et al. (2020) of the CCPS model (Loop 1 and 2) and the loop for FL interaction (Loop 3).” But should be, “Three loops were analysed based on the analysis of Chevalier et al. (2020) of the CCPS model (Loop 1 and 3) and the loop for FL interaction (Loop 2).” The corrected paragraph appears below.

In answering the fourth research question, sequential behaviour analysis was executed to examine the differences between the learning behaviours of the two groups. The behavioural sequence reaches a significant level (*p* < 0.05) when the *Z* value is more than 1.96 (*Z* > 1.96; Bakeman and Gottman, 1997). Figures 6, 7 present the behavioural transition diagrams of the students involved in two learning groups; the z-scores are shown on the middle line and each line's direction represents its transfer direction. Three loops were analysed based on the analysis of Chevalier et al. (2020) of the CCPS model (Loop 1 and 3) and the loop for FL interaction (Loop 2).

In addition, there was also an error in Results, Learning behaviours, Paragraph 4. This paragraph previously stated: “Otherwise, the ESL's three significant behaviour sequences are” but it should be “Otherwise, the EFL's three significant behaviour sequences are”. The corrected paragraph appears below:

Otherwise, the EFL's three significant behaviour sequences are: AT → CD, PM → ID, and LI → PLI. When aiming to reach the intended destination they demonstrated Loops 1 and 3. The EFL students collaboratively generated ideas by working on algorithms (AT → CD), and they physically expressed their ideas using gestures individually to justify their CT concepts to their partners (PM → ID). Such formulation fell into the essence of negotiation on problem-solving strategies, and thus the EFL students revealed their behaviours of significantly engaging in target-language interaction in Loop 2 (LI → PLI). Following the PP task of coding and conversation practice, the EFL students frequently interacted with one another, kept concentrating on the robots' movements, and used assigned English sentences when it was their turn. If errors occurred, the teacher would come by and guide them to use the taught sentence in their interaction (LI → PLI).

The authors apologize for these errors and state that this does not change the scientific conclusions of the article in any way. The original article has been updated.

## Publisher's note

All claims expressed in this article are solely those of the authors and do not necessarily represent those of their affiliated organizations, or those of the publisher, the editors and the reviewers. Any product that may be evaluated in this article, or claim that may be made by its manufacturer, is not guaranteed or endorsed by the publisher.
