# Corrigendum: Effects of a pair programming educational robot-based approach on students' interdisciplinary learning of computational thinking and language learning

**DOI:** 10.3389/fpsyg.2023.1229357

**Published:** 2023-06-28

**Authors:** Ting-Chia Hsu, Ching Chang, Long-Kai Wu, Chee-Kit Looi

**Affiliations:** ^1^Department of Technology Application and Human Resource Development, National Taiwan Normal University, Taipei City, Taiwan; ^2^Faculty of Artificial Intelligence in Education, Central China Normal University, Wuhan, China; ^3^National Institute of Education, Nanyang Technological University, Singapore, Singapore

**Keywords:** Interdisciplinary activities, educational robots, pair programming, language learning, trial-and-error loops

In the published article, there was an error in one of the *p*-values stated in the Results section.

A correction has been made to *Results, Learning Anxiety, Paragraph 2*. The corrected paragraph is shown below.

Table 5 presents that the five subscales of learning anxiety in the post-questionnaire differed significantly between the two groups (Wilks' lambda = 0.408, *F* = 7.26, *p* < 0.001, Eta = 0.59). The Bonferroni method was then used to analyse the confidence intervals. The results of the *post hoc* comparison indicated that the EFL group showed lower learning anxiety than the CSL group for the dimensions of speech anxiety, communication apprehension, and fear of being negatively evaluated by other students.

The authors apologize for this error and state that this does not change the scientific conclusions of the article in any way. The original article has been updated.

